# Supplementary figures and images for: NLRP3 inflammasome activation mediates sleep deprivation-induced pyroptosis in mice
Source: PeerJ. 2021 Jul 6;9:e11609. doi: 10.7717/peerj.11609 (PMC8269641; doi:10.7717/peerj.11609)

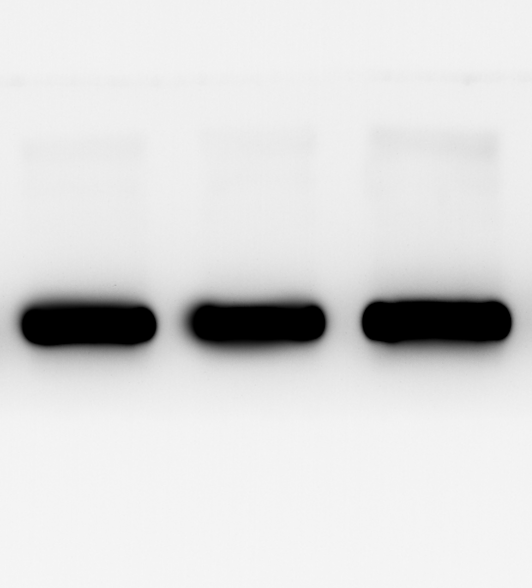

Supplement: Supplemental Information 1 [file peerj-09-11609-s001.zip › Weatern Blot raw data/Figure 2b actin.tif]

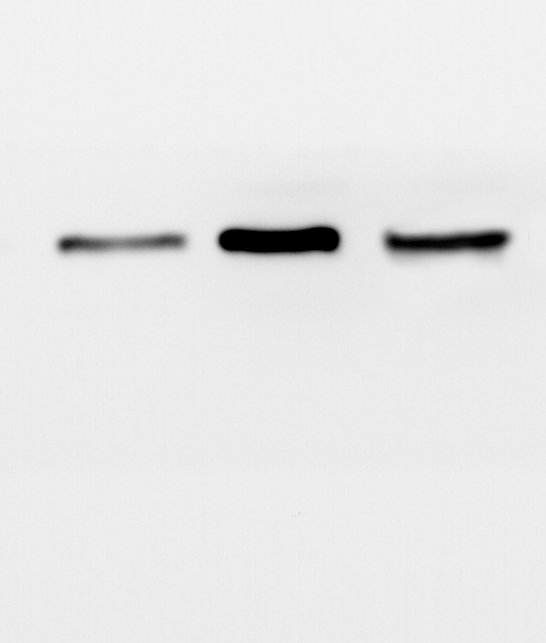

Supplement: Supplemental Information 1 [file peerj-09-11609-s001.zip › Weatern Blot raw data/Figure 2b ASC.tif]

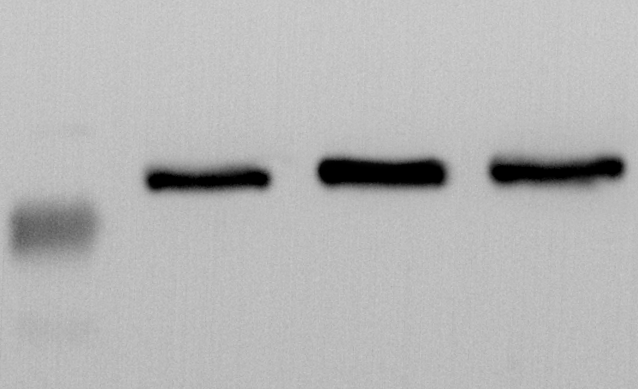

Supplement: Supplemental Information 1 [file peerj-09-11609-s001.zip › Weatern Blot raw data/Figure 2b caspase1.tif]

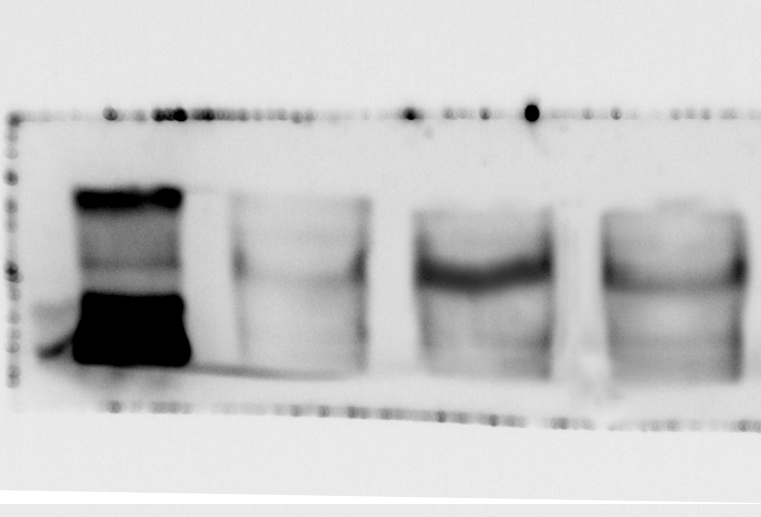

Supplement: Supplemental Information 1 [file peerj-09-11609-s001.zip › Weatern Blot raw data/Figure 2b NLPR3.tif]

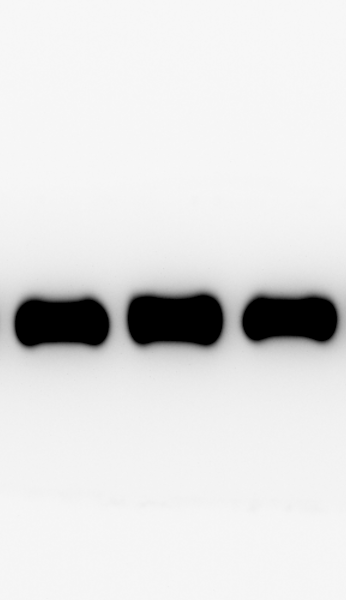

Supplement: Supplemental Information 1 [file peerj-09-11609-s001.zip › Weatern Blot raw data/Figure 3b actin.tif]

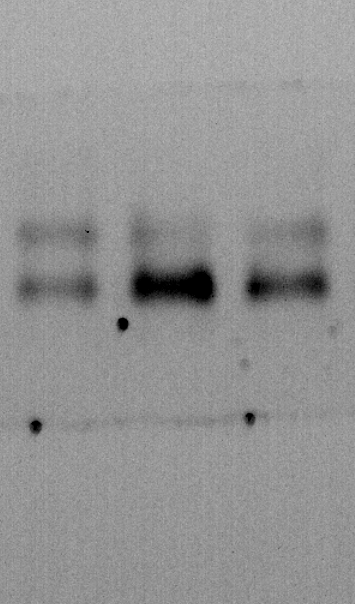

Supplement: Supplemental Information 1 [file peerj-09-11609-s001.zip › Weatern Blot raw data/Figure 3b GSDMD.tif]

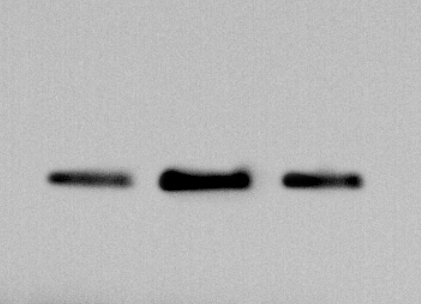

Supplement: Supplemental Information 1 [file peerj-09-11609-s001.zip › Weatern Blot raw data/Figure 4a P-p38.tif]

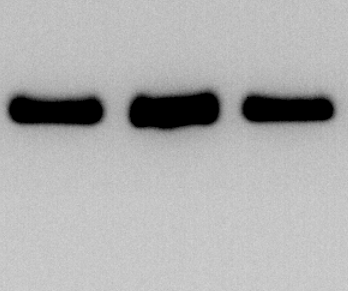

Supplement: Supplemental Information 1 [file peerj-09-11609-s001.zip › Weatern Blot raw data/Figure 4aT-p38.tif]

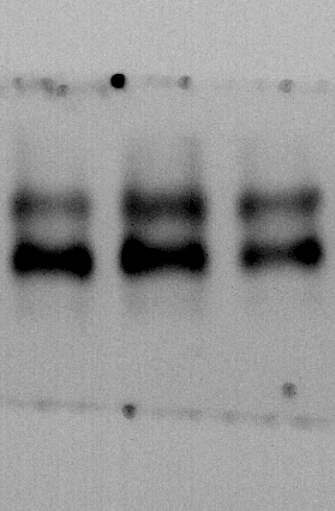

Supplement: Supplemental Information 1 [file peerj-09-11609-s001.zip › Weatern Blot raw data/Figure 4b p-erk.tif]

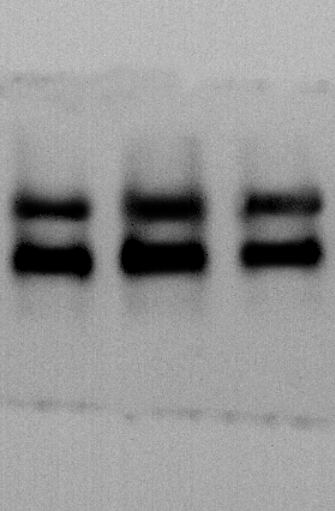

Supplement: Supplemental Information 1 [file peerj-09-11609-s001.zip › Weatern Blot raw data/Figure 4b T-ERK.tif]

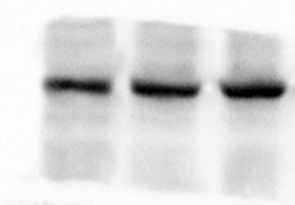

Supplement: Supplemental Information 1 [file peerj-09-11609-s001.zip › Weatern Blot raw data/Figure 4c p-AKT.tif]

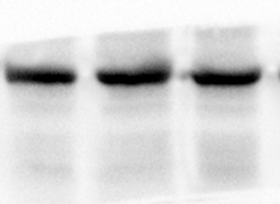

Supplement: Supplemental Information 1 [file peerj-09-11609-s001.zip › Weatern Blot raw data/Figure 4C T-AKT.tif]
